# Vascular calcification: Molecular mechanisms and therapeutic interventions

**DOI:** 10.1002/mco2.200

**Published:** 2023-01-03

**Authors:** Wei Pan, Wei Jie, Hui Huang

**Affiliations:** ^1^ Department of Cardiology, the Eighth Affiliated Hospital Sun Yat‐sen University Shenzhen Guangdong China; ^2^ Joint Laboratory of Guangdong‐Hong Kong‐Macao Universities for Nutritional Metabolism and Precise Prevention and Control of Major Chronic Disease Sun Yat‐sen University Shenzhen Guangdong China

**Keywords:** endoplasmic reticulum stress, mitochondrial dysfunction, molecular mechanism, therapeutic interventions, vascular calcification

## Abstract

Vascular calcification (VC) is recognized as a pathological vascular disorder associated with various diseases, such as atherosclerosis, hypertension, aortic valve stenosis, coronary artery disease, diabetes mellitus, as well as chronic kidney disease. Therefore, it is a life‐threatening state for human health. There were several studies targeting mechanisms of VC that revealed the importance of vascular smooth muscle cells transdifferentiating, phosphorous and calcium milieu, as well as matrix vesicles on the progress of VC. However, the underlying molecular mechanisms of VC need to be elucidated. Though there is no acknowledged effective therapeutic strategy to reverse or cure VC clinically, recent evidence has proved that VC is not a passive irreversible comorbidity but an active process regulated by many factors. Some available approaches targeting the underlying molecular mechanism provide promising prospects for the therapy of VC. This review aims to summarize the novel findings on molecular mechanisms and therapeutic interventions of VC, including the role of inflammatory responses, endoplasmic reticulum stress, mitochondrial dysfunction, iron homeostasis, metabolic imbalance, and some related signaling pathways on VC progression. We also conclude some recent studies on controversial interventions in the clinical practice of VC, such as calcium channel blockers, renin–angiotensin system inhibitions, statins, bisphosphonates, denosumab, vitamins, and ion conditioning agents.

## Graphical abstract

Vascular calcification is caused by the imbalance of vascular microenvironment. The pro‐calcific factors include chronic inflammation, endoplasmic reticulum (ER) stress, mitochondrial dysfunction, iron homeostasis, programed cell death, and other cellular metabolic dynamics. However, the pharmacological and nutritional modulations, such as CCBs, ACEI/ARB, and vitamins, may provide potential therapeutic strategies on vascular calcification.

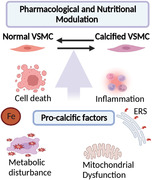

## 1 INTRODUCTION

**FIGURE 1:**
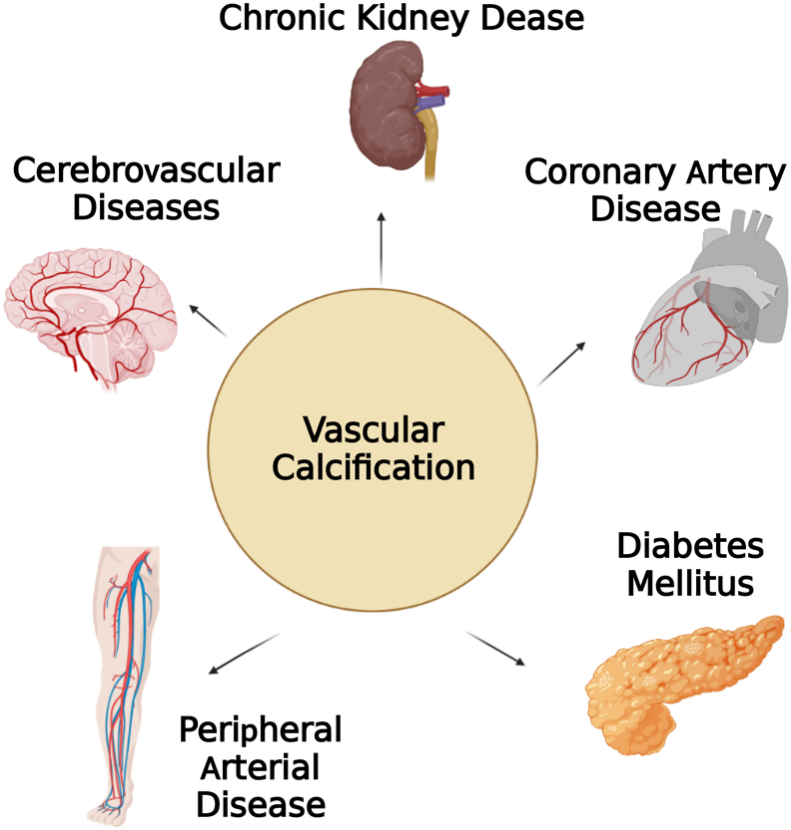
Multiple diseases related to vascular calcification (VC). VC participate in a lot of pathological process of several chronic diseases, such as cerebrovascular diseases, peripheral arteria diseases, and coronary artery disease (CAD)

Vascular calcification (VC) is defined as a calcium phosphate deposition process in cardiovascular diseases.^1^ Previous studies have emphasized advancing age as a main cause leading to the structural and functional changes in the vasculature.^2^ Besides aging, VC is closely related to the pathological process of several chronic diseases, such as atherosclerosis, hypercholesterolemia, diabetes mellitus (DM), hypertension, chronic kidney disease (CKD) and coronary artery disease, peripheral arterial disease^3^, ^4^, ^5^, ^6^, ^7^, ^8^ (as shown in Figure 1). In particular, it is a strong predictor of cardiovascular mortality rate for CKD patients.^9^ Recently, epidemiological studies have confirmed that higher scores on coronary artery calcification (CAC) measures are associated with higher incidence and mortality from coronary events, especially in patients with CKD.^10^, ^11^, ^12^

However, as every cardiologist knows, the regular operation of percutaneous coronary intervention could not solve CAC due to the increased vessel stiffness.^13^ Until now, no therapeutic strategy has been shown to be effective in reversing the progress of VC. As a result, scientists are doing their best to explore possible mechanisms underlying VC that may prevent it at an early stage and improve its prognosis.

VC was an extremely prevalent and complex biological process involving complicated pathophysiological mechanism. The accepted mechanism of VC‐included metabolic alterations induced calcium (Ca) and phosphate (P) abnormality,^14^ loss of calcification and mineralization inhibitors,^15^ osteogenic transdifferentiation of vascular smooth muscle cells (VSMCs) to calcifying vascular cells,^16^ products of matrix vesicles that act as a nidus of calcium phosphate deposition,^17^ nucleation of apatite formed by the release of apoptotic bodies or necrotic debris,^18^ deposits calcium phosphate hydroxyapatite caused by abnormal mineral homeostasis,^19^ formation of ectopic mineralization and nucleation complexes due to bone remodeling,^20^ and matrix degradation or modifications.^21^, ^22^

Recent studies have additionally explored the underlying cellular and molecular mechanisms during the above processes. In this review, we focus on the relationship of VC and underlying cellular and molecular mechanism, such as chronic inflammation, endoplasmic reticulum (ER) stress (ERS), mitochondrial dysfunction, iron homeostasis, programed cell death (PCD), as well as other cellular metabolic dynamics.^23^, ^24^, ^25^, ^26^, ^27^, ^28^ In addition, we collected the latest studies on the signaling pathways of VC to explore the possible molecular mechanism. In summary, we have reviewed the most recent research advances on VC to provide a better understanding of their pathogenesis and their relationship. We also reviewed the pharmacological and nutritional modulations of VC. Only with advanced understanding will effective therapeutic options for the prevention and treatment of VC be possible.

## 2 MOLECULAR MECHANISMS OF VC

### 2.1 The molecular mechanism of inflammation on VC

Inflammation is one of the most fundamental and essential immune reactions of the organism when facing infection, injury, or other noxious conditions.^29^ It is driven by a complex set of mediators, such as oxidation, carbonyl stress, C‐reactive protein (CRP), and various cytokines.^30^ However, inflammation can also cause undesirable responses, which induce exacerbating tissue damage and hazardous substances away from productive purposes.^29^ Therefore, recent scientific research has advanced the concept that chronic inflammation as a primary risk factor underlying aging and age‐related diseases.^31^ Continuous upregulation of pro‐inflammatory cytokines (e.g., tumor necrosis factor alpha [TNF‐α], interleukin [IL]‐1β, IL‐6, cyclooxygenase [COX]‐2, inducible nitric oxide synthase) can activate the downstream inflammatory signaling pathways to promote the progression of diseases.^32^, ^33^ Previous studies have found that VC can be regulated by inflammation in myriad aspects.

#### 2.1.1 The role of inflammatory cytokines on VC

As a complication of age‐related disease, inflammatory status can worsen the secretion of inflammatory factors to promote the progress of VC.^34^ It is today acknowledged that moderate chronic inflammatory status exacerbates diseases like osteoporosis, Alzheimer's disease, atherosclerosis (AS), and type 2 DM (T2DM).^35^ The individuals aged 60 and older with higher levels of inflammatory cytokines, such as IL‐1β, IL‐6, IL‐18, and TNF‐α, were more prone to developing CKD.^36^ In a prospective observational study among hemodialysis (HD) patients, malnutrition and inflammation were defined as low serum albumin (<40 g/L) and elevated high‐sensitivity CRP (hs‐CRP) (≥28.57 nmol/L) and were significantly associated with abdominal aortic calcification (AAC); the odds ratio of hs‐CRP was 1.561.^37^ A follow‐up of 30,703 consecutive individuals who were enrolled for CAC detection at a median of 79 months received the same conclusion that the greater CAC burden was associated with worse outcomes in the CKD patients (estimated glomerular filtration rate (eGFR) <60 ml/min/1.73 m^2^) only in those with higher hs‐CRP.^38^ Reducing systemic inflammation including the levels of circulating CRP, IL‐6, and neutrophil gelatinase‐associated lipocalin (NGAL) by the switch from 30 bicarbonate HD to mixed online hemodiafiltration can improve endothelial cells angiogenesis and reduce VC.^39^ IL‐17A was proved to significantly promote VC in an ex vivo aorta culture system detected by micro‐computerized tomography system, and the effect was concentration dependent.^40^ Numerous studies confirmed that macrophages, monocytes, lymphocytes, and other inflammatory cells can regulate VC by infiltrating plaque and releasing cytokines respectively.^41^, ^42^, ^43^ The study reported that vitamin D deficiency–related macrophage recruitment and further inflammatory response were involved in the process of VC.^44^ Diverse studies revealed that neutrophil/lymphocyte ratio can be used as a tool for assessing the severity of arterial stiffness.^45^ The cytoplasmic contents of exosomes released from dendritic cells contain a number of proteins and miRNAs that participate in vascular repair processes and calcification.^46^ Some of the inflammation‐related biomolecules are proposed to be the independent parameters for cardiovascular mortality in CKD such as cluster of differentiation (CD)40; CD154 were also reported greatly expressed at early stages of CKD vessels.^47^

#### 2.1.2 Inflammation triggers VSMCs osteogenic transformation

Several lines of evidence proved that inflammatory responses trigger and precede osteogenic differentiation of VSMCs.^27^ It is fully accepted that osteogenic transdifferentiating of VSMCs plays a pivotal role in regulating the remodeling processes of the VC.^48^ The phenotype shift of VSMCs from contractile type to osteo/chondrogenic type is promoted by the formation of calcifying vesicles, decrease of mineralization inhibitory molecules, and the functional shift of matrix.^16^, ^49^, ^50^ TNF‐α induced phenotypic changes and osteogenic transformation of VSMCs via the upregulation of Pit‐1 in aortic allograft rat calcification model.^51^ Sánchez‐Duffhues et al. also found that TNF‐α and IL‐1β induced endothelial‐to‐mesenchymal transition in human primary aortic endothelial cells, sensitizing the bone morphogenetic protein type II receptor (BMPR)2–C‐Jun‐N‐terminal kinase (JNK) signaling axis for osteogenic differentiation.^52^ There is study found that palmdelphin (PALMD) promoted calcification development via the VSMCs osteogenic conversion while PALMD gene silence inhibited the above process.^53^ In vitro molecular mechanism experiment proved that PALMD exerted the above effect through an adjustment of glycolysis and nuclear factor‐κB (NF‐κB)‐mediated inflammation.^54^ Therefore, we propose that targeting PALMD‐mediated glycolysis may represent a novel therapeutic strategy for treating calcification.

#### 2.1.3 Inflammation regulated the process of hydroxyapatite deposition

As we know, the hallmark of VC is abnormal deposits of hydroxyapatite mineralization. Inflammation regulates the process of mineralization by numerous pathways. For example, the transformed VSMCs induced by inflammation could produce excessive levels of matrix metalloproteinases, which in turn facilitate the growth of CaP crystal.^55^ Previous studies have recognized tissue‐nonspecific alkaline phosphatase (TNAP) as a pivotal enzyme responsible for skeletal or dental mineralization participating in VC.^56^ Recent studies have proved that TNAP as a typical enzyme exerting the anti‐inflammatory effect to dephosphorylate adenosine nucleotides and lipopolysaccharide.^57^

#### 2.1.4 Inflammation‐related molecules on VC

Multiple inflammatory pathways shared the common signaling pathway with ERS to initiate and aggravate VC. For example, Nod‐like receptor protein 3 (NLRP3) inflammasomes can be activated by ERS to induce inflammatory response via oxidative stress (OS), calcium homeostasis, and NF‐κB activation that result in the pathological process of VC.^58^ Inflammation can also reduce fetuin‐A, which is an essential serum protein synthesized in the liver to inhibit calcification.^59^

To sum up, experts pointed out that both systemic and local inflammation were crucial in the pathogenesis of VC.

### 2.2 How does ER stress influences VC

ER is a highly influential organelle for protein folding and maturation in organic cells during the process of protein package.^60^ ERS occurs when the ability of protein folding oversteps the capacity of ER.^61^ When ERS is overwhelming, the cell will be compelled to death.^62^ That is because ERS triggers an adaptive response of the cell, which is named unfolded protein response (UPR), which helps cells cope with the stress.^63^ A state of mild or moderate ERS in UPR, the homeostatic sets in motion transcriptional and translational changes that are beneficial to cell adaption and survival.^64^ However, a state of persistent ERS in UPR signaling is emerging as a pivotal contributor to an increasing list of human diseases, including VC, pulmonary hypertension, atherosclerosis, metabolic disease, and cancer.^65^, ^66^, ^67^ Hence, it is interesting to target signaling components of UPR as potential therapeutic strategies to combat these ERS–associated diseases. There are studies that have shown that ERS can promote the progress of VC through a variety of signaling pathways. Thereby, we discuss the mechanism of ERS on VC in this review.

#### 2.2.1 ERS regulated the phenotype transformation of VSMCs

ERS was a significant inducer for the osteogenic phenotype transformation of VSMCs to promote VC. As we know, VSMCs is a vital cell type involved in the progress of VC.^68^ The osteoblast conversion of VSMCs plays a key role in orchestrating calcification in the vasculature.^16^ This process accompanied with the loss of smooth muscle cells (SMCs) markers (SM22α and SM α‐actin) and gain of osteochondrogenic markers (Runt‐related transcription factor 2 [Runx2], bone morphogenetic protein [BMP]‐2, osteopontin, osteocalcin, and alkaline phosphatase [ALP]).^69^ Recently, three UPR pathways, inositol‐requiring enzyme 1 (IRE1)–X‐box binding protein 1 (XBP1), protein kinase RNA‐like ER kinase (PERK)–eIF2alpha (eIf2α)‐activating transcription factor‐4 (ATF4), and ATF6, play vital roles in the process of SMC transformation.^70^ There was a study found that IRE‐1–XBP1–glucose‐regulated protein 78 (GRP78) pathway was involved in the ERS‐mediated VSMCs differentiation and calcification through linking to the Runx2 promoter.^71^ Unspliced XBP1 could affect the expression of the osteogenic markers Runx2 and msh homeobox 2 (Msx2) by bounding directly to β‐catenin to influence the progression of VC.^72^ ERS can be enhanced due to the upregulation of the protein levels of ATF4 and osteoblast markers (osteopontin and osteocalcin),^73^ together with the downregulation of contractile markers (smoothelin, calponin, and SM22α), which resulted in the conversion of the VSMCs from contractile phenotype to osteogenic phenotype. Vice versa, when the levels of the contractile markers increased, the osteoblast‐like markers decreased, and VC is retarded with ATF4 knockdown.^74^ In vitro stearate‐induced VC mouse aortic vascular smooth muscle cells (MOVAS)‐1‐murine models, ATF4 regulates osteogenic differentiation and mineralization via PERK–eIF2α–ATF4–C/CCAAT enhancer binding protein β (EBP) homologous protein (CHOP) axis.^75^ However, most of the mechanisms of ATF4 regulating osteogenic phenotype transformation of VSMCs are indirect; it needs further study to explore the direct mechanisms.

#### 2.2.2 ERS mediates the release of EVs in VC

Extracellular vesicles (EVs) are typically classified into apoptotic bodies, exosomes, large‐size EVs (LEVs), and so on based upon their size, mechanisms of formation, and release pathways.^76^, ^77^, ^78^ Some initial studies have revealed that ERS might regulate the development of VC via EVs.^79^ Furmanik et al. showed the role of LEVs in VC induced by ERS. In in vitro treatment of VSMCs with tunicamycin and thapsigargin (ERS inducers), the releases of EVs were significantly increased, accompanied with the upregulation of sphingomyelin phosphodiesterase 3 (SMPD3) (an influential regulator for EV release). The effect was blocked when VSMCs were treated with SMPD3 inhibitor. These results suggest EVs are involved in ERS–induced calcification.^80^

Thus, deepening our understanding of the noxious signals carried by EVs in pathological situations characterized by prolonged activation of ERS is critical to identify novel specific therapeutic or preventative targets against VC.

#### 2.2.3 ERS regulate apoptosis in VC

ERS can also mediate apoptosis to participate in the progress of VC.^81^, ^82^ Apoptotic bodies, which have similarities with matrix vesicles, result in the local accumulation of calcium phosphate and provide an applicable microenvironment for the nucleation of calcium phosphate crystals.^83^ However, the precise molecular mechanisms of ERS‐mediated apoptosis in VC have not been completely elucidated. A recent study reported that IRE1α may act as an indispensable factor in ER‐induced calcium homeostasis disturbance inducing apoptotic cellular death via the inositol‐1,4,5‐trisphosphate receptor.^81^ Therefore, ER‐induced cell death is the key process contributing to VC. Previous studies suggested that ERS‐mediated PCD promoted VC due to the loss of calcium homeostasis^5^ or by increasing secretion of Grp78‐loaded EVs. EVs released from ERS‐stimulated VSMCs showed an increase in the Grp78 levels. Grp78/Grp94 deposition was aggrandized in the extracellular matrix of calcified arteries.^80^ There are studies proved that caspase 12‐dependent apoptosis promoted the initiation of ERS‐mediated apoptotic processes in calcified arteries. Transcriptional induction of C/EBP‐homologous protein also played an important part in calcified arteries.^84^ ERS‐mediated apoptotic processes are closely associated with the transcriptional inducer of CHOP and/or activation of JNK. CHOP is the most widely explored biomarker in ERS‐induced apoptosis signaling pathway in cardiovascular diseases.^85^ It is a leucine zipper transcription factor that induces cell apoptosis through the downregulation of the anti‐apoptotic protein Bcl‐2.^86^ Under chronic ERS, PERK activation can lead to the phosphorylation of eIF2α, which further promotes ERS‐induced apoptosis through modulating the translation of ATF4 mRNA and the induction of CHOP.^87^ ATF6 and XBP1 can also enhance the expression of CHOP.^88^ Caspases (cysteine‐dependent aspartate‐specific protease) are a family of cysteine proteases that play an essential role in cell apoptosis.^89^ Caspase‐12 in this superfamily has been proved to be activated by ERS.^90^ Functional caspase‐12 can activate caspase‐9 directly, which activates another caspase enzyme caspase‐3 to eventually lead to apoptotic process due to the prolonged ERS.^91^, ^92^ There is a study found that caspase‐12 expression was increased markedly by almost threefold in aortic calcified tissue in a rat model treated with vitamin D3 and nicotine; meanwhile, the CHOP level was significantly increased by about 10‐fold in the rapid calcification model which is treated with high‐dose vitamin D3. However, the JNK protein level of in the above models did not show statistical differences from that of controls.^84^ Hao et al. also got a similar result that caspase‐12 and CHOP were significantly upregulated in aortic calcified rat model.^93^ Fibroblast growth factor 21 exerts its inhibitory effect on VC by ameliorating ERS‐induced apoptosis, aortic calcium content, and ALP activity via CHOP and caspase‐12 pathways.^94^ Besides, activated RIE1α and TNF‐receptor‐associated factor 2 forms a complex network with the apoptosis signal‐regulating kinase 1 that mediates ERS‐induced cell apoptosis via the activation of JNK and p38 mitogen‐activated protein kinase (MAPK).^95^, ^96^ However, current evidence indicates that CHOP and caspase‐12 pathways are more significant in the progress of VC by alleviating ERS‐mediated VSMCs apoptosis. JNK may not be the major pathway of apoptosis in VC rats, which needs more research to prove.^93^, ^94^

#### 2.2.4 ERS is involved in the autophagy mediated VC

Previous studies have proved that ERS may be involved in the autophagy‐mediated VC. According to recent studies, autophagy was related to three arms of ERS and was activated as a protective mechanism to relieve unfolded proteins load.^97^, ^98^ For instance, active ATF‐4 has been reported to increase the transcription of autophagy‐related genes like Beclin‐1, autophagy‐related 3, autophagy‐related 12, and microtubule‐associated protein 1 light chain 3.^99^ Besides, cleaved ATF‐6 was proved to enhance the expression of death‐associated protein kinase 1 (DAPK1), which phosphorylates Beclin‐1 directly to regulate autophagy.^100^ Moreover, activated IRE‐1α led to the activation of MAPK, which is critical for the induction and regulation of autophagy.^101^ Autophagy can inhibit VC by reducing matrix vesicle release and Klotho pathway.^102^, ^103^ There is also a study found that VC may be alleviated due to the negative feedback of ERS and autophagy. Li et al. found that levels of ERS markers (GRP78, CHOP) and autophagy markers (LC3II and Beclin‐1) were increased in the calcified aorta of rats. Interestingly, 3‐methyladenine (an autophagy inhibitor) not only prevented autophagy but also promoted ERS in the calcified aorta and exaggerated VC.^104^ This study indicated that though ERS accelerated autophagy in the process of VC, autophagy decelerated ERS and calcification in a negative feedback mechanism. But it still needs further clarification.

#### 2.2.5 ERS bridges ferroptosis and VC

The mRNA sequencing result revealed that the suppressing exchange of cystine–glutamate activates an ERS response and ferroptosis, providing the potential relationship between ERS and ferroptosis.^105^ Further study on the molecular mechanism involved in the ERS responses, we found some shared molecules involved both in the process of VC and ferroptosis. A study on dihydroartemisinin (DHA) has found that ERS can be initiated by DHA in glioma cells, leading to the increase of HSPA5 expression and PERK‐upregulated ATF4. The upregulation of HSPA5 increased the expression and activity of an enzyme, which is the key regulator of ferroptosis, named glutathione peroxidase 4 (GPX4). The expressions of RUNX2, osterix, ALP, bone sialoprotein, and OPG in VSMCs, which are the characteristic markers of VC, were all elevated after ERS induced by PERK/ATF4.^106^ These results indicated that ERS response played a pivotal role in the cross talk between ferroptosis and VC via PERK/ATF4 axis. Besides, higher expressions of caspase‐3, Wnt1, galectin‐3, and BMP‐2 markers are present as the inducer for higher calcification content after ERS stimulation.^107^ BMP‐2 is proved to promote VSMCs calcification through the IRE‐1–XBP1– Grp78 pathway,^80^ whereas Grp78 was found to increase when ferroptosis of pancreatic cancer cells occurred after artesunate treatment.^108^ These results have revealed that BMP2/Grp78 is another molecular pathway stimulating ERS to induce ferroptosis and VC. ERS UPR was reported to take part in the pathogenesis of VC because the mechanistic target of rapamycin (mTORC) 1 activity was stimulated in the uremic state^109^ and PI3K–AKT–mTOR signaling axis prevented cancer cells from ferroptosis induction.^110^ Besides, ferroptotic agents were reported to activate UPR via the ERS‐mediated PERK–eIF2α–ATF4–CHOP–P53 upregulated modulator of apoptosis signaling pathway that is proved to contribute to TNF‐α‐induced VC.^73^ These data provided the evidence that ERS creates the linkage between ferroptosis and VC.

#### 2.2.6 The role of OS and VC

OS generates because of the overload of reactive oxygen species (ROS) production and the lack of antioxidant protection.^111^ A number of studies have shown that OS may contribute to the pathogenesis of VC progression^112^, ^113^; the underlying mechanism has not been fully elucidated. It has been proved that ERS is closely associated with OS, a major contributor to VC in human coronary artery SMCs.^70^, ^114^ Liu et al. also demonstrated that ERS homeostasis influences OS‐mediated VC.^115^

**FIGURE 2:**
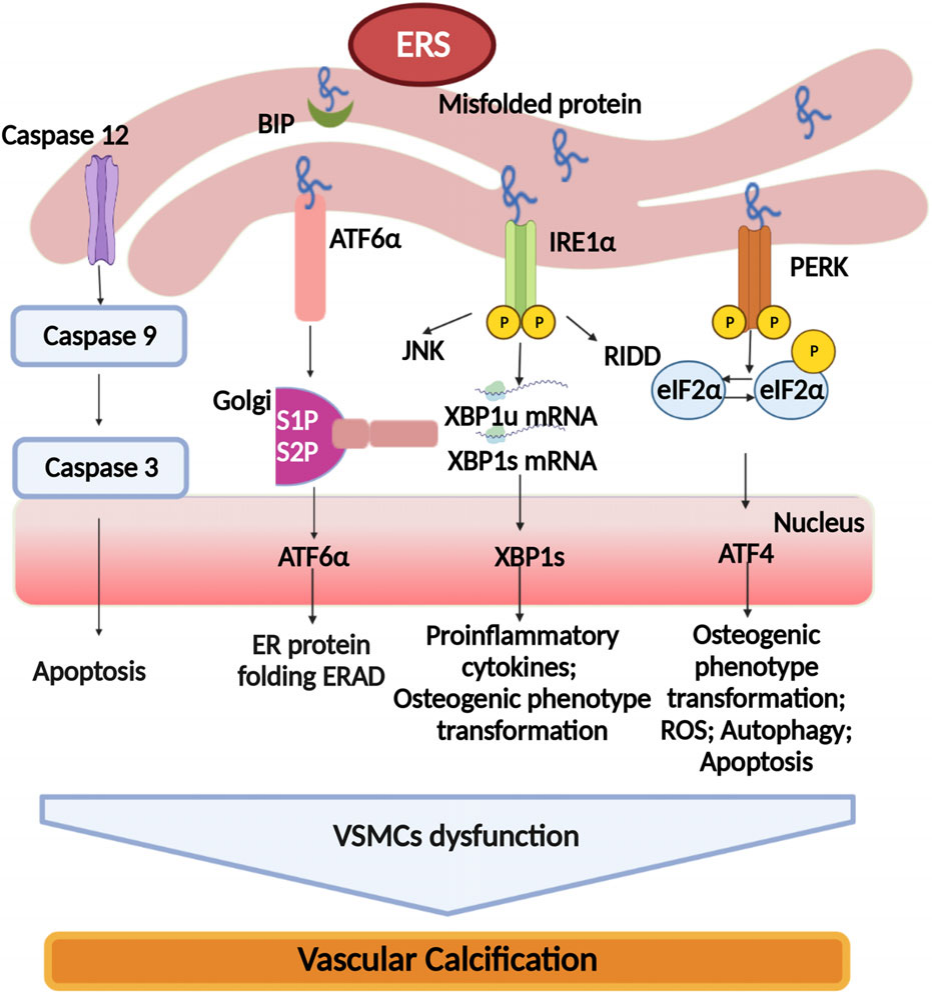
Influence of endoplasmic reticulum stress (ERS) on vascular calcification. Protein kinase RNA‐like endoplasmic reticulum kinase (PERK), inositol‐requiring enzyme 1 (IRE1), activating transcription factor‐6 (ATF6), and caspase signaling pathway promote vascular calcification (VC) by mediating unfolded protein response (UPR). Under unstressed circumstance by which PERK, IRE1, and ATF6 are combined with Grp78/BIP in inactive state. During pathological stimulation or unfolded/misfolded proteins accumulate in the endoplasmic reticulum (ER) luminal, the ER resident protein separate from these transmembrane protein sensors and further activate the UPR. Activated PERK phosphorylates eIF1α and promotes the protein translation. Reversely, ATF4 expression is increased. Phosphorylated IRE1 promotes the splice of X‐box binding protein 1 (XBP1) mRNA, leading to the XBP1 production. In addition, separated ATF6 is translated into the Golgi apparatus where it is cleaved by the proteolysis. Cleaved ATF6 subsequently moves into the nucleus and springs for the transcription of ER chaperones as well. Finally, caspase‐12 is activated by ERS. Functional caspase‐12 directly activates caspase‐9, which activates caspase‐3 in turn and eventually leads to apoptosis. These respective downstream processes of the four sensors accelerate VC of vascular smooth muscle cells (VSMCs)

In a summary, ERS prompts the progress of VC through complex molecular mechanisms and different signaling pathways (as shown in Figure 2).

### 2.3 The role of mitochondrial dysfunction on VC

**FIGURE 3:**
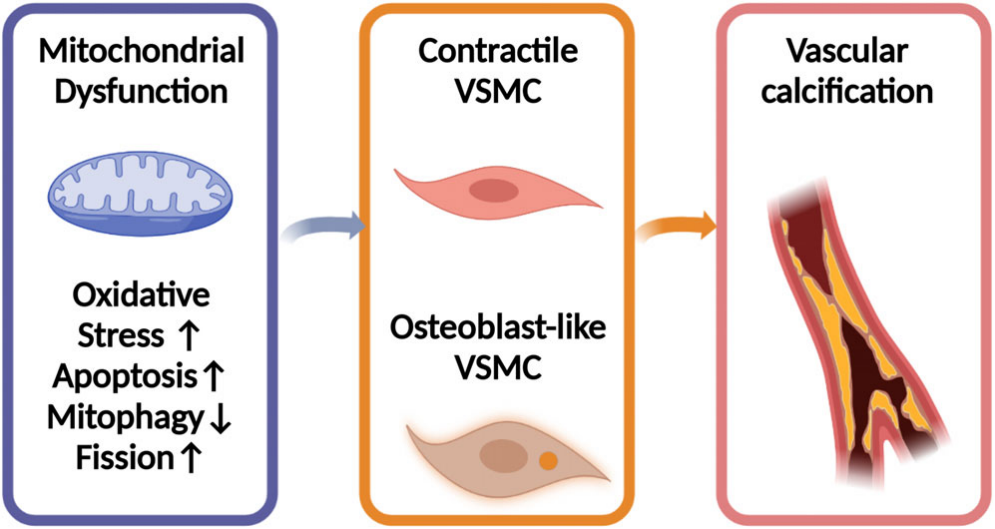
Mitochondrial dysfunction plays a key role in the progression of vascular calcification (VC). Oxidative stress, apoptosis, excessive fission, and rare mitophagy have been reported to damage the normal structure of mitochondria and impair respiratory function, resulting in the transformation of vascular smooth muscle cells (VSMCs) from contractile to osteoblast‐like cells and, therefore, VC‐mediated

Mitochondria play an important part in energy production, cellular metabolism, neoplasm, and aging.^116^, ^117^ ROS stimulated by OS results in a subsequent cascade of caspase activation, the ATP depletion, mitochondrial depolarization, the increase of mitochondrial permeability transition pores, and finally, the malfunction of mitochondria and cellular apoptotic death.^118^ Numerous studies reveal that VC is related to mitochondrial dysfunction. Excessive fission and scarce mitophagy were reported to damage the normal structure of mitochondria and impair aspiratory function, which was determined by methyl‐4‐phenyl‐1,2,3,6‐tetrahydropyridine (mPTP) opening frequency, mitochondrial membrane potential and its morphology under TEM, the generation of ATP, and the measurement of oxygen consumption rate. This process can be reversed by nuclear receptor subfamily 4 group A member 1 (NR4A1) silencing. However, NR4A1/DNA–PKcs/p53 pathway activation accelerated the osteoblastic phenotype transition of VSMC and calcium deposition.^119^ Therefore, protective mitophagy inhibits mitochondrial dysfunction and delays the progression of VC. For example, niclosamide was reported to protect against VC through targeting carnitine *O*‐octanoyltransferase mechanism, which exerts the function by mediating mitochondrial dysfunction and promoting the metabolic process of fatty acid.^120^ Attenuating neuronal mitochondrial dysfunction can be a useful target for treating neurodegenerative diseases at the presence of CKD due to VC. A recent study found that quercetin can attenuate VC by inhibiting mitochondrial fission.^121^ When the HG‐HUVEC‐Exos‐induced mitochondrial dysfunction was inhibited by the knockdown of the gene such as VCAN, the calcification and senescence of VSMCs can be prevented.^122^ Not only quercetin but also α‐lipoic acid can attenuate VC via the reversal of mitochondrial dysfunction and restoration of the Gas6/Axl/Akt survival pathway.^123^ One mechanism of the influence of mitochondrial dysfunction on VC is the response to the calcium and phosphate overload that drives senescent VSMCs into a pro‐inflammatory state.^124^ Based on all these literature, mitochondrial dysfunction was treated as a critical cause of VC^125^ (as shown in Figure 3).

### 2.4 The relationship between iron homeostasis and VC

Iron is an essential transition metal that participates in a lot of biological activities of cells.^126^ Though the emerging role of iron homeostasis in the regulation of bone development and metabolism has been clarified by numerous clinical trials.^127^ In the past, contradictory results produced by preclinical and clinical made it tough to conclude the definite effect of iron on VC. The “Iron hypothesis” was first raised in 1981 by Dr. Jerome Sullivan, suggesting that higher levels of stored iron promote cardiovascular diseases, whereas iron deficiency may have an atheroprotective effect. The “FeAST” trial has proved that there were highly significant reductions in all‐cause mortality and in combined death plus nonfatal myocardial infarction and stroke in association with iron reduction therapy.^128^ However, iron supplementation appears to be crucial as a therapy for CKD with anemia that is associated with VC. This “paradox” attracts a lot of studies pursuing the effect of intracellular macrophage iron on VC. Recently, abundant studies have discovered that iron homeostasis can affect the process of inflammation, ERS, and mitochondria function to regulate VC.

#### 2.4.1 Iron modulates inflammatory responses in VC

Iron is an essential micronutrient for many enzymatic reactions, which modulates the progress of inflammation.^129^ The iron levels contained in the macrophages are directly linked to the immune function of macrophages by the catalyzing of oxidative low‐density lipoprotein (LDL).^130^ Moreover, macrophage iron can increase the release of TNF‐α, IL‐6, and the potent monocyte–attracting chemokine monocyte chemoattractant protein‐1, and so on.^129^ The relationship between iron and inflammation is mutual. Iron utilization capacity can be reduced under the inflammatory status in CKD because of the increased levels of ferritin and hepcidin.^131^ Nowadays, both intravenous iron therapy and oral iron therapy have been applied in CKD patients to treat iron deficiency anemia.^132^

#### 2.4.2 Iron influences ERS to affect VC

Considering ERS, the evidence that there is a functional junction between ERS and iron metabolism came from studies on the genetic disorders of iron overload. When the load/capacity ratio of ER is broken by pathogenic states and exogenous alterations, UPR can be launched. ER homeostasis can be maintained not only under control of the interplay between UPR and major histocompatibility complex‐I but also the capacity of the C282Y faulty HFE to provoke UPR activation. Biological relevance of UPR‐induced modulation is also related to the iron homeostasis that opens a novel promising avenue in the research between ERS and iron homeostasis.^133^ Recently, a study has revealed that inflammation and ERS synergistically downregulated the expression of butyrate dehydrogenase type 2 in human THP‐1 cells, upregulated the expression of the iron carrier protein NGAL, and the stress‐inducible heme degrading enzyme heme oxygenase‐1 (HO‐1), which is a contributing factor for intracellular iron homeostasis resulting in the iron liberation.^134^

#### 2.4.3 Iron overload impairs mitochondrial function to induce VC

Iron overload caused by superfluous iron accumulation in the cardiovascular system was also an independent factor that impaired mitochondrial dynamics and led to the ferroptosis of cardiomyocytes.^135^ Mitochondria, as a vital organelle, not only provides the cells with ATP but also utilizes cellular iron to synthesize cofactors, including heme and iron–sulfur clusters.^136^ Because iron bioavailability is crucial for mitochondrial metabolism and biosynthesis, iron homeostasis is essential for mitochondrial function and dynamics. The transmembrane protein neuropilin‐1 (NRP1) promotes mitochondrial function in endothelial cells by preventing iron accumulation and iron‐induced OS through a VEGF‐independent mechanism.^137^ In turn, proper mitochondrial function is crucial to cellular iron homeostasis.^136^, ^137^ Iron overload cardiomyopathy is one of the typical diseases caused by iron imbalance–induced impaired mitochondrial dynamics which led to deterioration in cardiac performance.^138^

As we mentioned above, chronic inflammation, ERS, and mitochondria dysfunction underpin the pathogenesis of VC. Therefore, these findings corroborate the importance of iron homeostasis for VC.

### 2.5 Cell death on VC

As we know, VSMCs play a pivotal role in the progress of VC. Loss of VSMCs via varieties of cell death can lead to the thinning of fibrous cap and formation of necrotic core formation and calcification.^5^ Nowadays, the concept of PCD contains apoptosis, necrosis, necroptosis, pyroptosis, autophagy, ferroptosis, and so on.^139^ NLRP3‐mediated VSMC pyroptosis was reported to take part in the molecular protective mechanism of irisin against VC.^140^ HASMCs calcification was reported to restain in Atg7−/− animal model after AGEs induction. Atg7 was recognized as an autophagy inhibitor, indicating that autophagy was an important mechanism in diabetes‐related VC.^141^

#### 2.5.1 Inflammation bridges between ferroptosis and VC

Ferroptosis is a newly discovered type of PCD in 2012, which is triggered by intracellular phospholipid peroxidation. The small molecule compound erastin is an acknowledged inducer of ferroptosis. Ferroptosis is closely related with iron homeostasis. The process starts with the accumulation of excessive iron and lipid peroxidation in the body and ends up with the oxidative damage of the cells.^142^ The latest studies have already discovered that ferroptosis takes part in the pathological process of varieties of diseases, such as neoplastic diseases^143^ and neurological disorders^144^ (Alzheimer's disease, Parkinson's disease, and Huntington's disease). Ferroptosis is also proved to be involved in the progress of CKD.^145^ Meanwhile, patients with CKD can develop the intima and the media types of VC; media calcification is more specific to CKD and is the exclusive form of VC observed in pediatric CKD patients. This interaction creates a potential bridge between VC and ferroptosis. If VC is regarded as the result of inflammation, ferroptosis can be considered the cause of inflammation. Current studies found that ferroptosis results in the initiation of inflammation in a lot of diseases. For example, in nonalcoholic steatohepatitis patients, the protein levels of pro‐inflammatory cytokines, including TNF‐α, IL‐1β, and IL‐6, were significantly increased with RSL‐3 treatment, a ferroptosis activator. This increase can be reversed with the treatment of ferroptosis inhibitors such as sodium selenite (GPX4 activator) or liproxstatin‐1 (ferroptosis inhibitor).^146^ Intervention with liproxstatin‐1 significantly reduced the Szapiel and Ashcroft scores via nuclear factor erythroid 2‐related factor 2 (Nrf2)/transforming growth factor‐beta (TGF‐β)1 pathway in the radiation‐induced lung fibrosis model^147^ that was also involved in the regulation of VC.^148^ Excessive production of inflammatory factors can promote the progression of diseases. Targeting ferroptosis can effectively prevent exaggerated inflammatory response. For example, the expression levels of angiotensin II type‐1 receptor (AT1R), IL‐6, IL‐1β, COX‐2, and GFAP in the astrocytes can be significantly elevated by external injuries, but this elevation can be greatly suppressed by the introduction of ferrostatin‐1 (ferroptosis inhibitor) in a dose‐dependent manner.^149^ Many molecules and signaling pathways related to inflammation cross both in calcification and ferroptosis. Toll‐like receptors (TLR)4 play an essential role when ferroptosis orchestrated neutrophil recruitment to injured myocardium and coronary vascular endothelial cells after cardiac transplantation.^150^ However, TLR4 contributes to oxidized LDL‐induced calcification of human VSMCs.^151^ TLR4/NF‐κB axis was considered important inflammatory signaling for both VC and ferroptosis‐induced inflammatory response. An in vitro study showed that curcumin prevented ferroptosis‐induced acute kidney injury in mice by reducing myoglobin‐mediated inflammatory responses and ROS production through the cytoprotective enzyme HO‐1 activation and TLR4/NF‐κB axis.^152^ Collectively, these results confirm that ferroptosis, as a form of iron‐dependent cell death, may be an initiating factor for inflammation or at least has the pro‐inflammatory effects. Considering the mutual relationship connected by inflammation, we hypothesize that ferroptosis may share the same inflammatory process contributing to VC.

#### 2.5.2 Mitochondria dysfunction bridges between ferroptosis and VC

When elucidating the function of mitochondria dysfunction on VC, we noticed that the trigger of mitochondria dysfunction on VC was highly closely related to redox reaction that was also the initial factor through the process of ferroptosis. It is well accepted that ferroptosis ends up with cellular mitochondrial dysfunction and toxic lipid peroxidation accumulation. The antioxidant transcription factor Nrf2, which has a strong impact on upregulating of the endogenous inhibitor of nitric oxide synthase, either directly or indirectly modulates ferroptosis and mitochondrial function.^153^ On one side, H_2_O_2_ treatment can induce ferroptosis within 3 h by upregulating aquaporin (AQP) expression, Fe^2+^ level, and lipid peroxidation; on the other side, H_2_O_2_ treatment can induce mitochondrial dysfunction by downregulating protein prohibitin 2 (PHB2). To conclude the result, we noticed that mitochondrial dysfunction is connected with ferroptosis under oxidative status after H_2_O_2_ treatment.^154^ In reverse, ferroptosis accounts for the molecular mechanism associated with mitochondrial dysfunction and toxic lipid peroxidation in cells, which plays a critical role in suppressing cancer, neurodegenerative diseases,^155^ Alzheimer's disease–induced cardiac anomalies,^156^ and so on. Therefore, we conclude that ferroptosis can potentially be acted as the target for VC therapy by the inhibition of mitochondrial dysfunction.

#### 2.5.3 The influence of iron homeostasis on ferroptosis and VC

The relationship between iron homeostasis and ferroptosis is acknowledged. The characteristic of ferroptosis is iron‐dependent lipid peroxidation, inactivation of the lipid repair enzyme GPX4, and accumulation of iron‐dependent ROS. Iron overload can promote ROS generation that stimulates OS via the Fenton reaction, thereby activating the key process to propagate ferroptosis.^157^ Various diseases, including liver fibrosis,^158^ Alzheimer's disease,^159^ and cancer^160^, were all detected with the abnormal iron homeostasis and ferroptosis. Ferroptosis and iron homeostasis exert potentially injurious results under conditions of ischemia/reperfusion (I/R) injury,^161^ myocardial infarction,^162^ heart failure,^163^ coronary artery angioplasty,^164^ or heart transplantation.^150^ With the exploration of ferroptosis, iron homeostasis has become a promising therapeutic strategy to pursue. Combining with the particularity and importance of iron homeostasis on ferroptosis, we believed that ferroptosis provided a novel insight into the molecular mechanistic study of VC.

As we mentioned above, VC and cell death shared a lot of commonalities among inflammation, ERS, mitochondria function, and iron hemostasis in both mechanistic and clinical studies.

### 2.6 Metabolic imbalance on VC

A large amount of evidences suggest that Ca–P metabolic imbalance accounts for the pathophysiology of VC.^165^ Excessive phosphate concentration and pyrophosphate deficiency is associated with Ca–P imbalance. ATP, which is the main source for energy supply and extracellular pyrophosphate may increase pyrophosphate concentrations to inhibit VC.^166^ A recent study considered that the molecular mechanism underlying inorganic phosphate (Pi)‐induced VC is related to plasmalemmal and mitochondrial phosphate transporters.^167^ The transporters mediated Pi influx increased the depolarization‐activated Ca^2+^ entry that led to Ca^2+^ and Pi overload and OS. Therefore, therapeutic strategies targeting transporters mediating plasmalemmal and mitochondrial Pi influx protect against VC.^167^ Deubiquitinating enzyme BRCC36 was reported to reduce the activity of phosphorylation of β‐catenin in the nucleus via interacting with β‐catenin directly, which is the primary effector protein of the Wnt signaling pathway, thereby inhibiting VC.^168^ Magnesium was recently discovered as an important calcification protein particle for the mineralization of the extracellular matrix. Redressing the magnesium imbalance may provide a promising novel clinical tool to prevent VC.^169^ *N*‐acetylcysteine displays antioxidant and anti‐calcification properties by generating hydrogen sulfide (H_2_S) and glutathione (GSH), which promoted osteoblast differentiation and bone formation.^170^ The calcium binding protein S100 family exerts its biological functions both inside and outside cells as a calcium sensor/binding protein, S100A11, one member of the S100 family, was reported to exert a critical role in VC.^171^ A case‐control study has used metabolomic profiling to reveal that arginine/proline metabolism may be a reliable mechanism to induce VC.^172^

## 3 MOLECULAR SIGNALING PATHWAYS ON VC

Molecular signaling pathways influence various cellular processes, such as metabolism, gene expression, or iron‐channel activity. In an in vitro VC study, there were 88 genes detected upregulated, whereas 59 genes were downregulated in transcriptome sequencing after erythropoietin (EPO) treatment.^173^ GATA6 transcription factor, BMP2, RUNX2, osteopontin (OPN), and osteocalcin (OCN) were recognized as genes associated with bone formation.^173^ Receptors interact with both external and internal molecules to trigger the downstream signaling pathways to regulate cell function. Here we displayed some vital molecular signaling pathways affecting the progress of VC. Many molecules including peroxisome proliferator–activated receptor γ coactivator 1‐α (PGC‐1α), aryl hydrocarbon receptor (AhR)/NF‐κB, Nrf2, HO‐1, BMP2, RUNX2, osterix, molecules involved in the MAPK and Notch signaling pathways; some autophagy effectors as well as many microRNAs are all investigated in the progress of VC.^174^

### 3.1 BMP and Runx2 signaling pathways on VC

Members of the BMP family are considered to play key role in the progression of VC. BMP exerts its biological effects by binding with BMP type I receptor A (BMPR1A).^175^ Interleukin enhancer binding factor 3 promoted VC by acting on the promoter regions of BMP2 and signal transducer and activator of transcription (STAT)1 to upregulate expression levels of BMP2 and downregulate STAT1 expression.^176^ Runx2 signaling pathway is an important molecular mechanism for VC; SIRT6 was reported to bound to and deacetylate Runx2 to prevent the osteoblast differentiation in cultured VSMCs from VC.^177^ Forkhead box transcription factor (FoxO1) was reported to increase the levels of its downstream target gene nuclear factor of activated T cells 3 (NFATc3) to upregulate the expression of the osteogenic marker Runx2 and promote VC.^178^ BMP2 and Runx2 were very important downstream signaling molecules that are involved in VC progression. BMP‐2 can act as the upstream signal molecule of Runx2. For example, leptin was reported to promote lower extremity artery calcification in T2DM by upregulating BMP2 and Runx2, which additionally switches the phenotypic of VSMCs via PI3K/Akt signaling pathway.^179^ It was reported that *Ginkgo biloba* extract (EGB761) could ameliorate warfarin‐induced aortic valve calcification through the inhibition of the BMP2‐medicated Smad1/5/Runx2 signaling pathway.^180^ MicroRNA (MiR)‐133a and miR‐204 were reported to downregulate Runx2 expression after BMP induced VC.^181^, ^182^

### 3.2 Wnt/β‐catenin signaling pathway on VC

Wnt/β‐catenin is another signaling pathway closely related with VC. The study found that Wnt inhibitor Dickkopf1 alleviates VC by promoting the degradation of PLD1, which decreases the formation and degradation of autophagosome.^183^ MiR‐126/Klotho/SIRT1 axis inhibits Pi‐induced calcification via regulating the Wnt/β‐catenin signaling pathway.^184^ B‐cell CLL/lymphoma 9 (BCL9) is acknowledged as a Wnt/β‐catenin transcriptional cofactor and was reported to be a key mediator to induce osteogenic transdifferentiation of human dental pulp stem cells under hypoxic conditions.^185^

### 3.3 AMP‐activated protein kinase (AMPK) signaling pathway on VC

The AMP‐activated protein kinase (AMPK)‐mediated signaling pathway plays a very important role in VC. For example, AMPK/tissue inhibitors of metalloproteinases 4 signaling cascade in VSMCs were involved in the progress of VC.^186^ Interestingly, the knockdown of AMPK by siRNA significantly reversed the anti‐calcification effects of metformin, resveratrol, and exendin‐4 and the reduction of RANKL in the calcified VSMCs.^187^ Inhibition of AMPK‐mediated cell deaths was observed in the regulation of aldosterone on high phosphate‐induced VC. Thereby, a lot of downstream molecules are under investigation. For example, AMPK/mTOR was proved to be associated with VC promoted by AGEs via the suppression of cell deaths from rat thoracic aorta culture.^188^ This association with VC was also reported in the management with metformin, liraglutide, adiponectin, and melatonin.^189^, ^190^, ^191^, ^192^ The AMPK/optic atrophy 1 pathway was related to the protective effect of melatonin against calcium deposition on VC.^193^ Recent studies have found that VC is also affected by the AMPK/insulin‐induced genes (INSIGs) pathway, which suppresses Pi‐induced Dlx5 and Runx2 expressions.^192^ DAPK3 and mitochondrial derived peptide MOTS‐c mediated by AMPK signaling pathway were reported to inhibit VC.^194^, ^195^ All these in vitro and in vivo evidence highlight AMPK as an important signaling pathway for VC.

### 3.4 PI3K–AKT signaling pathway on VC

The phosphatidylinositol 3‐kinase (PI3K)–AKT signaling pathway is another bridge to regulate VC. Sirtuin (Sirt)1 was reported to efficiently blunt the inhibitory effect of intermedin on VC when cells were preincubated with inhibitors of PI3K.^196^ As we know, the osteoblastic differentiation of VSMCs is one of the most important cytopathologic mechanism of VC. Recent study has proved that glucagon‐like peptide‐1 exerted multiple cardioprotective functions such as regulation of osteoblastic transformation and calcification of VSMCs through the activation of PI3K‐AKT signaling.^197^ Meanwhile, an inhibition of the PI3K–AKT signaling axis can sensitize cancer cells to induce ferroptosis.^198^ This sensibility requires the help of mTOR that is also the important downstream molecule of AMPK signaling pathway.^110^ In xenograft mouse models for PI3K‐mutated breast cancer and PTEN‐defective prostate cancer, the combination of mTORC1 inhibition with ferroptosis induction resulted in near‐complete tumor regression. Therefore, the hyperactive mutation of PI3K–AKT–mTOR signaling protects cancer cells from OS and ferroptotic death. Besides mTOR, Nrf2 and p53 were also mediated by PI3K–AKT signaling pathway,^199^ indicating their critical role in mediating the ferroptotic response and VC. From this experiment, we could read Nrf2 and mTOR signaling molecules were involved in the progress of VC. The lysosomal‐CER–mTOR signaling was reported to accelerate arterial medial calcification.^200^ Nrf2/HO‐1 signaling pathway is Nrf2's most classic approach to playing roles.^201^ It is closely related to NF‐κB signaling pathway.^202^ Recently, various advances have been made to research and explain that by manipulating Nrf2/HO‐1 pathway, several diseases, such as cardiovascular disease,^203^ bronchopulmonary dysplasia,^204^ and neurotoxicity,^205^ can be prevented. A lot of studies have already given evidence that Nrf2/HO‐1 signaling pathway was involved in both VC and ferroptosis.^206^, ^207^ Recently, Metformin has been reported to attenuate hyperlipidemia‐associated VC through anti‐ferroptotic effect. This result provides direct evidence to elucidate the relationship between ferroptosis and VC, which brought more concern on the study of VC and ferroptosis.^208^

### 3.5 NOTCH signaling pathway on VC

NOTCH signaling pathway is a recent discovered calcification‐related pathway. NO has been identified as a calcification inhibitor and exerted its function through the activation of NOTCH signaling pathway, An proteomic approach has proved that *S*‐nitrosylation of ubiquitin‐specific peptidase 9, X‐linked (USP9X) could deubiquitinate and stabilize MIB1 for NOTCH signaling pathway activation during the progress of VC.^209^

### 3.6 Other signaling pathways on VC

As an important signaling molecule in inflammation, NF‐κB exerts a critical part. The study found that SIRT1 inhibits VC by reducing the activity of NF‐κB to prevent VSMCs senescence and osteogenic transition, which indicates the relationship between senescence and VC.^210^, ^211^ VC can be regulated via diverse mechanisms under different inductions. For example, a study found that atmospheric PM2.5 exposure stimulated the inflammatory responses and contributed to the incidence of VC through the OPG/receptor activator of nuclear factor‐kappa B ligand (RANKL) pathway activation. Moreover, PM2.5 induced ROS generation that also contributed to the consequence of VC.^212^

### 3.7 MicroRNAs and VC

MicroRNAs also take part in VC, reticulocalbin‐2 (RCN2), which promotes VC and exerts its effect by Reticulocalbin‐2 (RCN2)/STAT3/miR‐155‐5p feedback loop.^213^ MiR‐223‐3p was found to target IL‐6/STAT3 signaling way to inhibit VC.^214^ As the vital particles transporters released from cells for intercellular communication, exosomes could regulate VC through a variety of interactive mechanisms.^215^ Recent studies revealed the role of exosomes extracted by VSMCs regulating the process of VC by miRNAs such as miR‐92b‐3p decrease the Krüppel‐like factor 4 expression to inhibit VC.^216^

### 3.8 RNA methylations on VC

RNA methylation has become a current research hotspot recently in the mechanism study of vascular diseases.^217^ For example, N6‑methyladenosine (m6A) modification is the most widespread type of internal posttranscriptional RNA methylation in eukaryotic cells.^218^The maladjustment of m6A modification was closely related to VC.^219^

## 4 POTENTIAL THERAPEUTIC STRATEGIES OF VC

Although VC is a critical contributor to cardiovascular all‐cause mortality rate, there is no effective method to reverse the progress of VC. Therefore, recent therapeutic strategies focus more on the inhibition or defer the progress of VC.^220^ Numbers of preclinical and clinical studies tried to find further evidence for the management of VC. We concluded some major clinical studies on VC therapy in Table 1. Pharmacological or Nutritional Modulation of VC were still under broad application. Here we discussed some advanced research on the drug to provide evidence in clinical application. The effective drug treatment includes calcium channel blockers, renin–angiotensin system inhibition, statins, bisphosphonates and denosumab,^221^ and SNF 472.

**TABLE 1:** Major preclinical and clinical trials of therapeutic strategies on vascular calcification (VC)

Study name	Type of study	Intervention/treatment	Condition or disease	Number of participants	Detecting method	NCT number	Phase
International nifedipine trial on antiatherosclerotic therapy (INTACT)^222^	Prospective, randomized, controlled trial	Drug: nifedipine, placebo	Coronary artery disease	426	Coronary angiography	–	Phase IV
International nifedipine GITS study intervention as a goal in hypertension treatment (INSIGHT)^223^	Prospective, randomized, controlled trial	Drug: nifedipine GITS, a thiazide diuretic	Hypertension	6592	Blood pressure detecting	–	Phase IV
Coronary artery calcification treatment with zocor (CATZ)^224^	Prospective, randomized, controlled trial	Drug: simvastatin placebo	Coronary artery calcification	80	Multidetector cardiac computed tomography	–	Phase IV
The St Francis Heart Study^225^	Prospective, randomized, controlled trial	Drug: atorvastatin, vitamin C, vitamin E, placebos	Asymptomatic patients with no prior history, symptoms, or signs of atherosclerotic cardiovascular disease (ASCVD)	5585	Electron‐beam computed tomography	–	Phase IV
Effectiveness of a proactive cardiovascular primary prevention strategy, with or without the use of coronary calcium screening, in preventing future major adverse cardiac events (CorCal)	Prospective, randomized, controlled trial (ongoing)	Drug: statin	Cardiovascular primary prevention strategy	9000	Coronary artery calcium (CAC) screening	NCT03439267	Not applicable
The multiethnic study of atherosclerosis (MESA)	Prospective, observational cohort trial	Drug: nitrogen‐containing bisphosphonate (NCBP) therapy	subclinical cardiovascular disease	6814	Computed tomography	NCT00005487	–
Study investigating the effect of drugs used to treat osteoporosis on the progression of calcific aortic stenosis. (SALTIRE II)^226^	Prospective, randomized, controlled trial	Drug: denosumab, alendronic acid, denosumab placebo, alendronic acid placebo	Calcific aortic stenosis	152	Quantity of calcium on the aortic valve measured using computed tomography	NCT02132026	Phase II
ADVANCE study^227^	Prospective, randomized, controlled trial	Vitamin D	CKD together with vascular and cardiac valve calcification	360	Agatston and volume scoring using multi‐detector computed tomography	NCT00379899	Phase IV
Vitamin K1 to slow vascular calcification in hemodialysis patients (VitaVasK trial)^228^	prospective, randomized, parallel group, multicenter trial	Vitamin K1	CKD with thoracic aortic and coronary artery calcification	384	multi‐slice computed tomography (MSCT)	NCT01742273	Phase III
The effects of vitamin K2 supplementation on the progression of coronary artery calcification (VitaK‐CAC)^229^	Prospective, randomized, parallel group, in two centers	Dietary supplement: menaquinone‐7 (vitamin K2), placebo capsules	Coronary artery disease	180	Multi‐slice computed tomography (MSCT)	NCT01002157	Not applicable
Vitamin K2 supplementation and arterial stiffness in the renal transplant population (KING)^230^	Prospective, single group study	Dietary supplement: vitamin K2 (MK7)	Arterial stiffness	60	Carotid‐femoral pulse wave velocity (cfPWV)	NCT02517580	Phase II
The effect of vitamin‐K 1 and Colchicine on vascular calcification activity in subjects with diabetes mellitus (ViKCoVaC)^231^	Prospective, 2 × 2 factorial trial	Vitamin‐K1, placebo	Diabetes mellitus and coronary calcification	149	^18^F‐NaF PET	^–^	^–^
Reversal Of arterial disease by modulating magnesium and phosphate (ROADMAP)^232^	Prospective, randomized, controlled trial	Magnesium citrate supplementation and phosphate‐binding therapy	Arterial stiffness in moderate chronic kidney disease	180	Repeated 18F‐FDG and 18F‐NaF PET‐scans	–	–
Effect of SNF472 on progression of cardiovascular calcification in end‐stage renal disease (ESRD) patients on hemodialysis (HD) (CaLIPSO)^233^	Prospective, randomized, controlled trial	Drug: SNF472, placebo	Cardiovascular diseases, cardiovascular abnormalities, calcifications, vascular end‐stage renal disease ESRD coronary artery calcification	274	Dual‐energy X‐ray absorptiometry (DXA) scans	NCT02966028	Phase II
Shockwave medical peripheral lithoplasty system study for PAD (Disrupt PAD III)	Prospective, randomized, controlled trial	Device: Shockwave Lithoplasty peripheral lithoplasty system drug: Medtronic IN.PACT (DCB)	Peripheral arterial disease	306	Angiography	NCT02923193	Not applicable
Effect of gemigliptin on biomarkers of kidney injury and vascular calcification in diabetic nephropathy: a randomized controlled trial^234^	Prospective, randomized, controlled trial	Drug: gemigliptin	Diabetic nephropathies	201	CAVI, CAC score (unit), CAVI normal, CAC score	NCT04705506	Not applicable

*Note*: The phase of “not applicable” describes trials without FDA‐defined phases, including trials of devices or behavioral interventions.

Abbreviations: CKD, chronic kidney disease; PAD, peripheral arterial disease.

### 4.1 calcium channel blockers and VC

Nifedipine is widely used as a typical calcium channel blocker. It exerts the inhibitory properties on VC by ameliorating glycation of LDL levels and decreasing the advanced glycation end products (AGE)‐induced receptor for AGE (RAGE) expression in endothelial cells through its anti‐oxidative effect especially in DM patients.^235^ A total of 201 patients with a total calcium score of ≥10 were recruited for the study of nifedipine on VC. The result revealed that the speed of coronary calcification in hypertensive patients was slower, indicating the potential prospect of nifedipine.^236^ The Tramadol (TRAM)‐34 is the specific KCa3.1 inhibitor that is the centralized conductance of calcium‐activated potassium channels expressed in various tissues. The experiment has found that TRAM‐34 reduced the activity of NF‐κB and TGF‐β signaling pathways thereby blocking the phenotypic transformation of VSMC and interfering with VC.^237^ Verapamil is a member of L‐type calcium channels that can inhibit VC by decreasing the ALP activity of SMCs and inhibiting the VSMCs mineralization and matrix vesicle activity.^238^ Azelnidipine was also reported to inhibit VC through reversing Msx2‐dependent osteogenic transition and matrix mineralization of VSMCs.^239^ Although the positive results were gained from the international nifedipine trial on anti‐atherosclerotic therapy (INTACT) trial^240^ and intervention as a goal in hypertension treatment (INSIGHT) trial,^236^ there is insufficient evidence to show that CCBs are able to significantly reverse the progression of CAC.^241^ With regards to the effect of CCBs on calcification of peripheral arteries, evidence for a positive effect still need further explored.

### 4.2 Renin–angiotensin system inhibition and VC

Angiotensin‐converting enzyme inhibitor and/or angiotensin II receptor blocker have been widely used in predialysis CKD patients. To explore the effect of renin–angiotensin system Inhibition, a retrospective single‐center observational study was implemented. A total of 121 predialysis CKD patients (age 71 ± 12 years; male 72 with eGFR 20.2 (11.8–40.3) ml/min/1.73 m^2^) who underwent thoracoabdominal plain computed tomography scan were enrolled in this study. The result has proved that angiotensin‐converting enzyme inhibitor (ACEI)/angiotensin‐receptor blocker (ARB) user is significantly and positively associated with low eGFR.^242^ The available intravascular ultrasonography studies on coronary arteries have proved that ARB such as olmesartan and telmisartan exerted a significant effect on plaque regression; however, the use of ACEI did not demonstrate such an effect.^243^ Notwithstanding these clinical data, the effect of ACEI/ARB on VC is still less clear.

### 4.3 Statins and VC

The effect of statins on VC attracts a lot of research. Theoretically, statins lower the levels of LDL‐cholesterol, which reduces the triggers for vascular inflammation and osteogenic differentiation of VSMCs.^244^ However, experiments on statins showed somewhat erratic results. There is study found that statins are capable of promoting atherosclerotic calcification through the disinhibition of a macrophage Rac1‐IL‐1β signaling axis.^245^ There is also a study reported that pravastatin can alter the microarchitecture of aortic calcium deposits to protect against VC.^246^ Clinical studies also show disparate results. The PARADIGM Study sought to elucidate the effect of statins on coronary atherosclerotic plaques. In a total of 1255 patients (60 ± 9 years of age; 57% men), 1079 coronary artery lesions were accessed. The result found that statin‐taking patients presented a slower rate on the progression of overall percent atheroma volume (PAV) but a higher rate on the progression of calcified PAV. Progression of noncalcified PAV and annual incidence of new high‐risk plaque features were lower in statin‐taking patients.^247^ Interestingly, a study on the effect of 80 mg simvastatin on coronary and abdominal aortic arterial calcium got the result that simvastatin exerted no effect on the progression of coronary artery calcium or AAC compared with placebo.^224^ Some trials even got the opposite result. Raggi et al. recruited 495 asymptomatic subjects with CAC for the study. Interestingly, for patients with standard therapy of statins, the progression of CAC was significantly greater when together with an MI event than in event‐free subjects.^248^ By comparing these data, we notice that the effects of statins on VC depend not only on the dosage, but also on the conditions of patients, especially the basal levels of LDL‐C and calcium scores, which indicates the progression in calcified plaques.^249^

### 4.4 Bisphosphonates and denosumab on VC

Bisphosphonates and denosumab were reported to affect the progress of VC via the suppression of atherosclerotic lesions and mineralization.^250^, ^251^, ^252^ However, the functions of bisphosphonates are disputable. Some experiments found that bisphosphonates can prevent from VC in several animal models through their affinity to hydroxyapatite.^253^, ^254^, ^255^ Bisphosphonates can directly inhibit VC in CKD patients independent of bone resorption. Inhibition of VC by local catheter‐based delivery of bisphosphonate zoledronic acid is proved effective without evident short‐term complications.^255^ Alendronate therapy significantly reduced mRNA levels of osteoblast‐related markers (Runx2 and TNAP) and ameliorated ALP activity and reduced the deposition of extracellular calcium in calcified VSMCs.^256^ A novel bisphosphonate compound named FYB‐931 was reported to preferentially prevent aortic calcification in vitamin D3–treated rats.^257^ However, there are also some studies trends in different directions. Bisphosphonates are reported to induce vascular inflammation and cause atherosclerotic plaque rupture.^258^ Among women in the different MESA cohorts, nitrogen‐containing bisphosphonates (NCBPs) exerted diverse effects on the participants at different ages. In elder subjects, NCBPs were observed to be related with a lower prevalence of cardio VC, whereas in younger subjects, NCBPs were related with a higher prevalence of cardio VC.^259^ A prospective, three‐center study has proved that denosumab and alendronate treatment improved lumbar spine bone mineral density, reduced bone turnover markers, and appeared to be effective for VC in HD patients with osteoporosis.^260^ Similar results were gained in alternative denosumab clinical trials.^226^, ^261^, ^262^, ^263^ The animal model revealed the protective effect of denosumab on VC may be associated with NF‐κB signaling pathway.^262^ The FREEDOM trial demonstrated the result that denosumab has no effect on the progression of aortic calcification or incidence of adverse cardiovascular events during 3‐year's observation in postmenopausal together with osteoporosis women who had a elevated cardiovascular risk.^264^ Though the above results provide some evidence on the application of bisphosphonates and denosumab, there are limitations among these trials. The sample sizes were small with a short duration of treatment, as well as the confounding factors need to be corrected; we still need further study for clinical use.

### 4.5 Vitamins on VC

Vitamins are extremely important supplementations for VC. Vitamin (Vit) K is one of the most widely used nutrients. The rationale for vitamin K to treat VC lies in the activation of extracellular matrix proteins, especially matrix G1a protein (MGP). As the carboxylated form of MGP acts as an inhibitor for VC and vitamin K is the cofactor of MGP, vitamin K deficiency can result in the reduction of available cMGP which led to VC.^265^, ^266^ Vitamin K is composed of various isoforms. The two main structures are Vit K1 and Vit K2. Vit K2 has been proved to be a bioactive compound in regulating the process of osteoporosis and inflammatory responses without risk of negative side effects or overdosing.^267^ Vit K2 exerts dual roles on the bone and vascular system. Vit K2 deficiency was typical of insufficient calcium deposition in the bone but high deposition in the vessel wall.^268^, ^269^ Recently, there is a study confirmed that Vit K2 might induce the autophagy of osteoblasts during the differentiation and mineralization.^270^ The Valkyrie Study explored the effect of vitamin K on VC progression in 132 subjects who were subjected to HD and atrial fibrillation prescribing with VKAs; the result concluded that although high‐dose vitamin K2 increased the vitamin K status in patients who underwent HD, there was no significant favorable effect observed on VC progression, indicating there is no significant causality between vitamin K status and VC progression.^271^ Although Vit K1 and Vit K2 are different in their absorption, the roles of Vit K1 and Vit K2 on VC are both protective. A large prospective randomized multicenter clinical trial named “VitaVasK” has proved that vitamin K1 supplementation effectively prevents the progression of coronary and aortic calcification in HD patients.^228^ A 1‐year vitamin K1 supplementation study in patients with aortic valve calcification confirmed that Vit K1 can lower the progression of calcification by 50% compared with placebo.^272^ Further prospective studies, including randomized controlled trials with larger number of participants, are necessary to evaluate whether vitamin K supplementation is beneficial for calcification. Other vitamins were also reported to suppress the progression of VC.^273^ For example, vitamin D deficiency can mediate VC while supplementation with vitamin D to adequate levels can accelerate perivascular adipose tissue macrophage infiltration and induce local inflammation, which further prevents VC.^44^ But the role of vitamin D on VC is still controversial. Some studies suggest that vitamin D is associated with VC. In a rat calcification model, the dosage of 7.5 mg/kg vitamin D plus nicotine can create the calcification situation with a range from 10‐ to 40‐fold increase in aortic calcium content and a decrease of medial elastic fibres.^274^ One mechanism of vitamin D mediated VC is associated with VSMC osteogenic differentiation and mineralization.^275^, ^276^, ^277^, ^278^ Considering the molecular mechanism of vitamin D on VC remains to be clarified, the clinical application of this double‐edged sword is still under investigation.

### 4.6 Iron supplementation on VC

As we mentioned above, iron hemostasis is a key regulator for the progression of VC. On one hand, iron deficiency causes anemia in CKD patients, the iron supplementation has already been prescribed to these patients.^132^, ^279^ Considering this, two oral formulations (iron citrate and sucroferric oxyhydroxide) have been approved for hyperphosphatemia treatment in CKD patients.^280^ On the other hand, iron overload contributes to the process of endothelial cell calcification by inducing apoptosis and ferroptosis.^281^ The mechanism underlying the protective effect of iron on VC is that iron regulated the Ca–P imbalance, which induced extracellular osteo‐chondrogenic shift of VSMCs.^282^ However, iron overload promoted ROS generation that enhanced the progress of VC.^283^ Recently, iron‐mediated ferroptosis of VSMCs was confirmed to promote VC, and this process can be inhibited by Metformin through Nrf2 signaling pathway.^208^ Therefore, the appropriate dose of iron supplementation for treatment should be confirmed, and further clinical studies should be required.

### 4.7 Magnesium and VC

Besides iron, the potential anti‐calcific roles of nutraceuticals (including magnesium, zinc, and phytate) are also reported. Magnesium contributes to various biological processes within the human body, such as muscle contraction/relaxation,^284^ immune responses,^285^ and bone formation.^286^ Recent studies have shown protective effect on cardiovascular diseases such as hypertension.^287^ Magnesium is known as a natural blocker for the calcium channel that regulates VC through maintaining Ca–P hemostasis.^288^, ^289^ It can also prevent VC by the inhibition of hydroxyapatite crystal formation.^290^ The mechanical study proved that the reversal effect of magnesium on high‐phosphate‐associated VC was associated with TRPM7 and Pit‐1.^291^ To better explore the protective effect of magnesium on VC, a randomized, controlled clinical trial enrolled 59 subjects who underwent HD for end‐stage kidney disease (ESKD) for intervention. The result revealed that magnesium decreased calcification propensity in subjects undergoing maintenance HD.^292^ Collecting all these data, we conclude that magnesium may become a promising therapy for VC.

### 4.8 Zinc and VC

Zinc is a novel discovered nutraceutical related to calcification. Zinc supplementation was found to ameliorate phosphate‐mediated osteo‐/chondrogenic transdifferentiation of VSMCs and protect against VC through GPR39–TNFAIP3–NF‐κB axis.^293^ This protective effect was also observed in high‐glucose as well as high‐phosphate state.^294^, ^295^, ^296^ Therefore, zinc supplementation may be a promising medicine to hinder the progress of VC in CKD.

### 4.9 Phytate and VC

Phytate is a potential nutrient for VC treatment.^297^ SNF472, which acts as an exogenous source of phytate, exerts an inhibitory effect on VC.^298^, ^299^, ^300^ The clinical trial proved that an adequate prescription of phytate can protect against AAC in CKD patients.^301^ Inositol hexakisphosphate (IP6, phytic acid), an endogenous compound existing widespread in mammalian cells or tissues, inhibits the mineralization of osteoblast cultures to protect against VC.^302^ More prospective studies must be performed to elucidate the benefits of a phytate‐rich diet and the associated risk of phosphorus bioavailability in these patients.^303^

### 4.10 Other novel findings on VC therapy

Recently, a newly discovered medicine named SNF472 has been approved to enter into the final stage of clinical trial. Considered a leading potential compound for the treatment of VC in ESKD‐stage patients, SNF472 exerts its function by targeting the hydroxyapatite deposited in the vascular wall directly. The present data from the trials have found that SNF472 is safe and effective for VC. However, more trials were implemented to collect the solid evidence for clinical use.^304^

Besides advances on the chemotherapy of VC, the emergence of intravascular lithotripsy (IVL) provides a neoteric choice for VC therapy.^305^ The available evidence has proved the effectiveness of this strategy. The long‐term benefit of IVL in VC application needs further human studies.

The effects of the above therapeutic strategy on VC from clinical trials are summarized in Table 2.

**TABLE 2:** Summary of drugs showing potential in treating vascular calcification (VC)

Agents	Condition or disease	Study type	Effects	References
Simvastatin	Coronary and abdominal aortic arterial calcium	Randomized controlled trial	No effect on VC	^224^
Vitamin K1	Hemodialysis	Randomized controlled trial	Inhibit VC	^228^
Nifedipine	Hypertension	Randomized controlled trial	Inhibit VC	^236^
Verapamil	High phosphate	In vitro cell model and in vitro rat aorta	Inhibit VC	^238^
ACEI/ARB	CKD	A retrospective single‐center observational study	Not clear	^242^
Atorvastatin	Atherosclerosis	In vitro cell model and in vivo rats model	Promote VC	^245^
Pravastatin	Hyperlipemia	In vivo mice model	Inhibit VC	^246^
Bisphosphonates	Atherosclerosis	In vivo mice model	Not clear	^250^
Pamidronate and etidronate	Renal failure	In vitro aorta and in vivo rats model	Inhibit VC	^253^
Vitamin D3	High nicotine	In vivo rats model	Promote VC	^274^
Iron citrate	High phosphate	In vitro cell model and in vivo rats model	Inhibit VC	^282^
Magnesium	High phosphate	In vitro cell model	Inhibit VC	^169^, ^290^
Zinc sulfate	High phosphate	In vitro cell model and in vivo mouse model	Inhibit VC	^293^
SNF472	Hemodialysis	Randomized controlled trial	Inhibit VC	^299^

Abbreviations: ACEI, angiotensin‐converting enzyme inhibitors; ARB, angiotensin receptor blockers; CKD, chronic kidney disease.

## 5 CONSLUSION AND PESPECTIVE

Despite a number of studies on the complexity and diversity of pathophysiological mechanisms in VC development caused numerous troubles in the discovery of the optimal drug targets, the exploration on VC has never stopped. In the past few decades, accumulating evidence has increased our knowledge of the pathogenesis of VC. As we know, the transition of VSMCs and hydroxyapatite deposition are primary processes in the progress of VC formation.^306^, ^307^ The concrete mechanisms underlying the processes are extremely complicated. In this review, we conclude some critical molecular mechanisms to influence the above processes, such as inflammation, ERS, mitochondria function, OS, iron homeostasis, and Ca–P metabolic imbalance. All these cellular mechanisms affect the progress of VC from diverse aspects. VSMCs phenotype transition and calcium deposition are two main targets to conquer for VC reverse.

**FIGURE 4:**
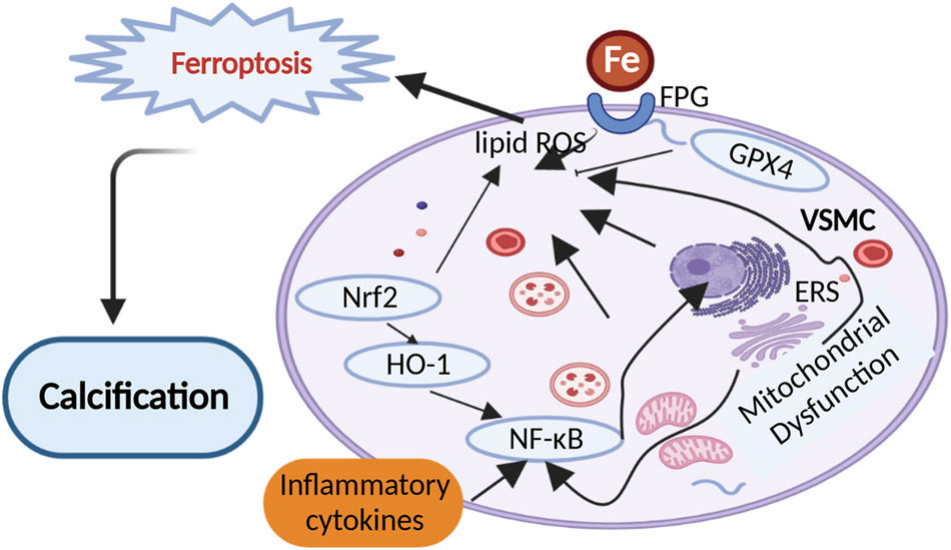
**Ferroptosis can be initiated by many factors to induce vascular calcification (VC)**. Mitochondria dysfunction, endoplasmic reticulum stress (ERS), inflammation, and iron homeostasis promote ferroptosis by initiating factor for inflammation or pro‐inflammatory effects, which contributes to vascular calcification

The abovementioned cellular mechanisms are reported to trigger the PCD in VC. Very recent evidence underscores the importance of ferroptosis on VC. Ferroptosis is recently reported as one type of iron‐dependent cell death responsible for many cardiovascular diseases.^308^ Our review summarized the recent experiments on the relationship between ferroptosis and VC.^139^, ^208^, ^281^ From the experiments, we could see that VC and ferroptosis shared several common signaling pathways in their initiation and development, indicating that ferroptosis may be also considered a key determinant in the progress of VC (as shown in Figure 4). Therefore, targeting ferroptosis might provide a different strategy for VC prevention or treatment. Although the existent evidence only rested on the laboratory stage, we still need more data to elucidate their relationship.

Interestingly, we noticed that not only VSMCs phenotype changes but also endothelial cell dysfunction exacerbates VC.^309^ In our review, we also displayed some recent experimental results connecting inflammation, ERS, mitochondria dysfunction, iron homeostasis, oxidative responses, and so on with endothelial dysfunction.^39^, ^281^

There are several signaling pathways or signal molecules mentioned related to VC. BMP2, Runx2, and Wnt/β‐catenin were known as critical biomarkers of VC. AMPK, PI3K/Akt, and NOTCH signaling pathways also contribute to VC. RNA modifications including RNA methylation as well as RNA network in VC have become a hot topic under investigation. Advances in epigenetic inheritance may promote the perception of VC.^310^

In the part of therapeutic strategies, we reviewed some recent pharmaceutical and nutritional advances on VC. Until now, HD seems to be the best choice to survive for CKD patients.^311^, ^312^ However, the side effects of HD, such as hypotension, electrolyte abnormalities, infection, fluid overload, as well as dialysis disequilibrium syndrome, in turn worsened the disease of CKD.^313^ Therefore, prevention before entering into the end stage via appropriate medicines is essential. Our review displayed the frequently used medicines applied in clinical practices like calcium channel blockers, renin–angiotensin system inhibitions, statins, bisphosphonates, and denosumab. They all presented some limitations because of the impaired metabolic capacity. The applications of vitamins, iron, zinc, magnesium, and phytate supplementations provide potential therapeutic strategy to prevent VC. However, all these nutritional supplementations can only play roles as assisting the mainstream treatment. The emergences of SNF472 and IVL technology bring new opportunities for VC therapy. More data need to be collected for clinical use. Therefore, there is still a long way to explore the appropriate therapy for reversing or curing VC.

To sum up, we reviewed some novel and controversial advances on the mechanical and therapeutic strategies that may provide new insights into future management of VC.

## AUTHOR CONTRIBUTIONS

Wei Pan, Wei Jie, and Hui Huang substantially contributed to discussion of the content and wrote, reviewed, and edited the manuscript before submission. All authors approved the final version of the manuscript.

## CONFLICT OF INTEREST

The authors declare that they have no conflict of interest.

## ETHICS STATEMENT

No ethical approval was required for this study.

## Data Availability

Not applicable.
